# Human Rotavirus Reverse Genetics Systems to Study Viral Replication and Pathogenesis

**DOI:** 10.3390/v13091791

**Published:** 2021-09-08

**Authors:** Satoshi Komoto, Saori Fukuda, Takayuki Murata, Koki Taniguchi

**Affiliations:** Department of Virology and Parasitology, Fujita Health University School of Medicine, Toyoake 470-1192, Japan; saorif@fujita-hu.ac.jp (S.F.); tmurata@fujita-hu.ac.jp (T.M.); kokitani@fujita-hu.ac.jp (K.T.)

**Keywords:** human rotavirus, reverse genetics, rescue T7 plasmid, helper expression plasmid, 11 plasmid-only system

## Abstract

Human rotaviruses (HuRVAs) are highly important causes of acute gastroenteritis in infants and young children worldwide. A lack of reliable and reproducible reverse genetics systems for HuRVAs has limited a proper understanding of HuRVA biology and also the rational design of live-attenuated vaccines. Since the development of the first reverse genetics system for RVAs (partially plasmid-based reverse genetics system) in 2006, there have been many efforts with the goal of generating infectious recombinant HuRVAs entirely from cloned cDNAs. However, the establishment of a HuRVA reverse genetics system was very challenging until 2019. This review article provides an overview of the historical background of the recent development of long-awaited HuRVA reverse genetics systems, beginning with the generation of recombinant human-simian reassortant RVAs with the aid of a helper virus in 2006 and the generation of recombinant animal (simian) RVAs in a helper virus-free manner in 2017, and culminating in the generation of recombinant HuRVAs entirely from plasmid cDNAs in 2019. Notably, the original HuRVA reverse genetics system has already been optimized to increase the efficiency of virus generation. Although the application of HuRVA reverse genetics systems has only just been initiated, these technologies will help to answer HuRVA research questions regarding viral replication and pathogenicity that could not be addressed before, and to develop next-generation vaccines and intestine-specific rotaviral vectors.

## 1. Introduction

Reverse genetics, a technology that enables the de novo synthesis of live (infectious) viruses from cloned cDNA(s), is a most instructive tool to understand multiple aspects of virus biology (e.g., viral replication, pathogenesis, and virus-host interactions), as well as to generate vaccine seed strains and viral vectors. Since the first generation of an infectious RNA virus with a single-stranded RNA (ssRNA) genome of positive-sense orientation, bacteriophage QB, in 1978, numerous reverse genetics technologies have been developed for the majority of important viruses in the human population. Common reverse genetics approaches permit the rescue of infectious recombinant viruses upon transfection of cDNA(s) into cultured cells [1]. However, RNA viruses with segmented genomes are relatively resistant to attempts to generate recombinant viruses entirely from cloned cDNAs due to the fact that multiple genomic RNAs must be supplied simultaneously in just a single cell [2].

Group A rotaviruses (RVAs) are non-enveloped, double-stranded (ds)RNA viruses belonging to the genus *Rotavirus* and family *Reoviridae*. RVAs are ubiquitous intestinal pathogens causing severe diarrhea in the young of many mammals, including humans, and avian species worldwide. An RVA virion contains an 18.6 kb 11-segment dsRNA genome, which encodes 11 or 12 viral proteins, six structural ones (VP1-VP4, VP6, and VP7), and five or six non-structural ones (NSP1-NSP5/6) [3]. All gene segments are mono-cistronic, with the exception of gene segment 11 that encodes NSP5 and NSP6 [3]. An RVA gene segment contains a central coding region that is flanked at the terminal ends by untranslated regions (UTRs), which serve as promoters to initiate genome replication and gene transcription by the viral polymerase [3]. The 11-segment genome is embedded in a virion comprised of three concentric capsid layers: the core particle composed of VP1 (RNA-dependent RNA polymerase, RdRp), VP2 (scaffolding function), and VP3 (capping enzyme); an intermediate layer of VP6; and outer layers of VP7 and VP4 [4,5,6]. The outermost VP7 and VP4 proteins contain the major antigenic determinants responsible for the induction of neutralizing immune responses, and define the G and P genotypes, respectively. Currently, at least 36 G and 51 P genotypes have been identified (https://rega.kuleuven.be/cev/viralmetagenomics/virus-classification/rcwg) (accessed on 10 July 2021).

Human RVAs (HuRVAs) are primary pathogens of childhood severe diarrheal disease and are associated with high morbidity and mortality. HuRVA gastroenteritis is associated with ~128,500–215,000 deaths each year in children <5 years of age [7,8]. Despite their medical importance, a lack of an efficient HuRVA in vitro culture system involving continuous cell lines and a HuRVA reverse genetics system had historically hindered a proper understanding of how HuRVAs replicate and induce severe gastroenteritis in humans.

Many animal RVAs can grow to high titers in cell lines; however, HuRVAs are more difficult to adapt to cell culture [9,10,11]. As a breakthrough as to this barrier in the HuRVA research field, the roller-tube culture technique was shown to be beneficial for adaptation of HuRVAs in cultured cells, presumably by increasing attachment to cells [12,13]. In spite of this success in HuRVA cultivation in cell culture, the viral propagation efficiency of representative HuRVAs (~10^6^ plaque-forming unit (PFU)/mL) is still very low compared to that of animal RVAs (~10^8^ PFU/mL) [14,15].

The other barrier in the HuRVA research field was the inability to develop a reverse genetics system for HuRVAs. The development of reverse genetics systems has been very difficult even for high-yield animal RVAs. The nucleotide sequences of all the genomic segments of simian RVA strain SA11 had been determined in 1990 [16]; however, the first RVA reverse genetics system was only described in 2006 [17], with which genetically modified recombinant RVAs can be generated with the aid of a helper virus (described in detail in Section 2). Subsequently, in 2017, an entirely plasmid-based reverse genetics system was developed for a high-yield animal RVA strain [18] (described in detail in Section 3). However, no HuRVA strain exhibiting high-yield growth potential in cell culture, analogous to simian RVA strains, was known; therefore, the development of a HuRVA reverse genetics system was more difficult than for animal RVA strains. Therefore, the generation of replicating HuRVAs entirely from cloned cDNAs remained the barrier that needed to be overcome to address a broad range of basic questions in HuRVA biology and applied questions in HuRVA disease, and to implement rationally attenuated HuRVAs as next-generation vaccines and rotaviral vectors of intestine-specificity. The following sections describe the key advances that led to the development of a prime HuRVA reverse genetics system, and further optimization of the HuRVA reverse genetics technology to increase the efficiency of virus generation.

## 2. Partially Plasmid-Based Reverse Genetics Systems for RVAs

In contrast to positive-sense ssRNA viruses, the naked genomic dsRNAs of RVAs alone do not initiate the production of infectious viruses when transfected into permissive cells. In the RVA replication cycle, a positive-sense ssRNA with a 5’ cap acts as both the mRNA and template for negative-sense ssRNA synthesis to yield genomic dsRNA [3]. Notably, genomic dsRNA synthesis occurs within a core particle that is assembled from newly synthesized VP1, VP2, and VP3 proteins, and positive-sense ssRNAs. A logical conclusion from these facts was that a cDNA-derived RVA positive-sense ssRNA supplied within an RVA-infected cell could be recognized by viral RdRp and then incorporated into the viral replication cycle to yield an infectious recombinant RVA possessing a cDNA-derived genomic dsRNA gene segment.

The breakthrough in genetic engineering of live RVAs came in 2006, when Komoto et al. (2006) [17] developed an RVA reverse genetics system to modify a single genomic segment with the aid of a helper virus. A plasmid cDNA encoding the full-length positive-sense ssRNA of the simian RVA VP4 gene of the P[2] genotype was cloned immediately downstream of the T7 RNA polymerase promoter, leading to the generation of a precise 5’ end when the plasmid was transcribed by the T7 RNA polymerase. To generate a precise 3’ end of the artificial RVA positive-sense ssRNA, the hepatitis delta virus (HDV) ribozyme and T7 RNA polymerase terminator sequences were inserted immediately downstream of the 3’ end of the RVA cDNA. Following T7 RNA polymerase-mediated transcription of the RVA-ribozyme hybrid RNA, the ribozyme sequence is removed through autocatalytic cleavage activity, thereby generating the exact 3’ end [19]. Thus, the structure of the RVA plasmid (rescue T7 plasmid) was designed to allow the de novo synthesis of full-length RVA positive-sense ssRNAs with the correct 5’ and 3’ ends. In addition, another advantage of employing T7 RNA polymerase was its functionality in a broad spectrum of cell types and its cytoplasmic localization [20], the cell compartment where RVA replication occurs. A recombinant vaccinia virus (VV) strain, rDIs-T7pol, expressing T7 RNA polymerase [21] was employed to supply the T7 RNA polymerase intracellularly. That is, prior to transfection of the rescue T7 plasmid coding for the VP4 gene segment of the P[2] genotype, COS-7 cells were infected with rDIs-T7pol. Next day, the infected and transfected cells were superinfected with a wild-type HuRVA of the P[8] genotype as a helper virus to provide the remaining 10 gene segments, i.e., other than the VP4 segment, and viral proteins required for RVA replication. The first infectious recombinant RVA, a reassortant virus containing 10 gene segments from a helper HuRVA and a cDNA-derived simian RVA VP4 gene segment of the P[2] genotype, was successfully recovered when lysates of transfected and superinfected COS-7 cells were passaged in MA104 cells in the presence of anti-VP4 neutralizing monoclonal antibodies of P[8]-specificity to suppress the growth of the helper virus. Thus, it was demonstrated that artificial RVA positive-sense ssRNAs can be not only transcribed and replicated, but also be packaged into a progeny virion.

By employing the original partially plasmid-based reverse genetics system and modifications of it, novel infectious recombinant RVAs with a cDNA-derived modified VP4 [14,22,23], NSP2 [24], or NSP3 [25,26] gene segment have been reported. In these helper virus-dependent reverse genetics systems, recombinant RVAs have to be selected against the vast background of helper viruses. The selection systems developed so far are based on antibody selection, siRNA selection, temperature sensitivity, and/or preferred packaging of several rearranged segments. Because targeted genetic manipulation of an RVA genome had been impossible until then, a tremendous advantage of the helper virus-dependent reverse genetics systems was that the cloned cDNA copy of an RNA gene segment allowed for the insertion of site-directed mutations into RVA genomes. These prime reverse genetics systems are technically demanding, resulting in recovery of live recombinant RVAs in low efficiency; however, they opened the door to the genetic engineering of infectious RVAs.

## 3. Entirely Plasmid-Based Reverse Genetics Systems for Animal RVAs

As described above, strong selection condition(s) to isolate a recombinant RVA from the vast majority of helper viruses have to be developed for partially plasmid-based reverse genetics systems, but such conditions have been developed for only three of the 11 gene segments (VP4, NSP2, and NSP3) over the past 15 years. Therefore, rotavirologists could not manipulate RVA genomes at will using the initial reverse genetics systems. However, several important lessons could be learnt from these primary systems, that is, intracellular synthesis of artificial RVA positive-sense ssRNAs with precise 5’ and 3’ ends from transfected rescue T7 plasmids was the key to the successful recovery of recombinant RVAs, suggesting that transfection of cultured cells with a set of 11 rescue T7 plasmids encoding a whole RVA genome could lead to the production of replicating RVAs in a helper virus-free manner. Thus, to overcome the limitation that helper virus-dependent reverse genetics systems are restricted to only three of the 11 genomic segments, rotavirologists devoted themselves to the development of a helper virus-free RVA reverse genetics system. Kanai et al. (2017) [18] reported a breakthrough in this research field, i.e., that an infectious recombinant animal (simian) RVA could be generated entirely from transfected plasmid cDNAs in cultured cells. Simian SA11-L2 virus [27] with the characteristic high-yield propagation in cell culture was the first RVA strain to be engineered entirely from cloned cDNAs. To generate a recombinant SA11-L2 virus, BHK-T7 cells, consistently expressing the T7 RNA polymerase, were transfected with a set of 11 rescue T7 plasmids encoding all 11 gene segments of SA11-L2 virus, together with three helper expression plasmids encoding the VV-capping enzyme subunits and small membrane fusion protein of bat orthoreovirus under the control of the eukaryotic promoter. Utilization of the capping enzyme and small membrane fusion protein was expected to enhance the recovery of recombinant viruses. Viable recombinant SA11-L2 virus could be recovered when the transfected BHK-T7 cells were co-cultured with and passaged in MA104 cells.

**Figure 1:**
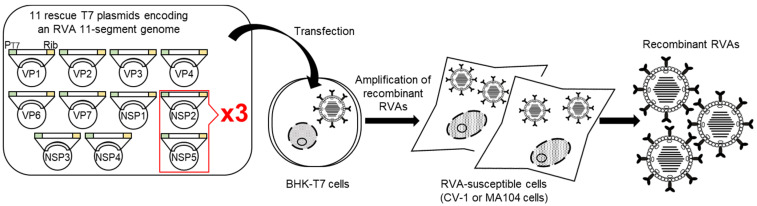
Schematic representation of the 11 plasmid-only reverse genetics system (Komoto’s reverse genetics system). BHK-T7 cells are transfected with a set of 11 rescue T7 plasmids encoding an RVA genome with 3-fold increased proportions of T7 plasmids for the NSP2 and NSP5 genes compared to the other nine T7 plasmids. Next day, the transfected BHK-T7 cells are overlaid with RVA-susceptible cells (CV-1 or MA104 cells) and then co-cultured for 3 days for amplification of recombinant RVAs. Infectious recombinant RVAs can be recovered from the co-cultures. PT7 and Rib denote the T7 RNA polymerase promoter and HDV ribozyme sequence, respectively.

Shortly after the development of this helper virus-free reverse genetics system, Komoto et al. (2017) [28] described that the use of two helper expression plasmids encoding the VV-capping enzyme is not essential for virus rescue. However, these new helper virus-free reverse genetics technologies required optimization because their efficiencies as to virus generation were insufficient for the rescue of attenuated recombinant RVAs by introducing site-directed mutations into viral genomes. Komoto et al. (2018) [29] reported a simple and robust entirely plasmid-based reverse genetics system, in which a helper expression plasmid is no longer required. Titration experiments to optimize RVA reverse genetics technologies revealed that higher amounts of two rescue T7 plasmids for the NSP2 and NSP5 genes have a positive impact on virus generation. In the optimized system, more highly efficient virus generation (>×10^5^) can be obtained following introduction into BHK-T7 cells of just a set of 11 rescue T7 plasmids encoding a genome of SA11-L2 virus with 3-fold increased amounts of two rescue T7 plasmids for the NSP2 and NSP5 segments (Komoto’s reverse genetics system; 11 plasmid-only system) (Figure 1). By using the 11 plasmid-only system, anyone can now routinely engineer infectious recombinant SA11-L2 virus efficiently just from a set of 11 rescue T7 plasmids. Thus, it has become possible to generate even severely attenuated recombinant RVAs that possibly would have not been possible with the original reverse genetics system. Because the 11 plasmid-only system is a simple and robust reverse genetics system, it has already been disseminated and reproduced in multiple research laboratories around the world for different research purposes. Using the 11 plasmid-only system and similar procedures based on it, in which the quantity ratio of two rescue T7 plasmids for the NSP2 and NSP5 segments is increased by 3-fold in relation to the other nine plasmids, successful generation of wild-type and several interesting mutant viruses with the genetic backbone of SA11-L2 virus has become possible.

**Figure 2:**
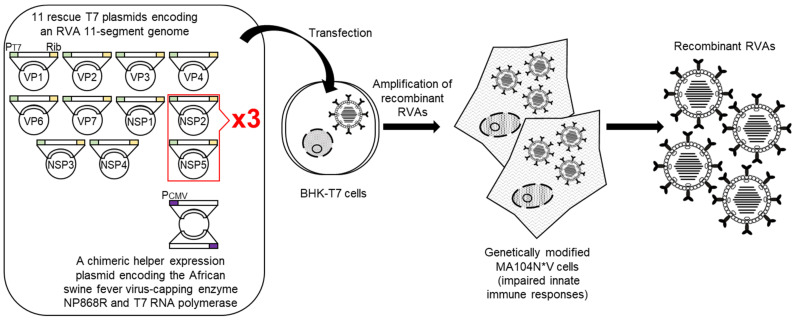
Schematic representation of Sánchez-Tacuba’s reverse genetics system. BHK-T7 cells are transfected with a set of 11 rescue T7 plasmids encoding an RVA genome with 3-fold increased proportions of T7 plasmids for the NSP2 and NSP5 genes compared to the other nine T7 plasmids, as well as a chimeric helper expression plasmid for the African swine fever virus-capping enzyme NP868R and T7 RNA polymerase. Next day, the transfected BHK-T7 cells are overlaid with genetically modified MA104N*V cells with impaired innate immune responses and then co-cultured for 3 days for amplification of recombinant RVAs. Infectious recombinant RVAs can be rescued from the co-cultures. PT7, Rib, and PCMV denote the T7 RNA polymerase promoter, HDV ribozyme, and cytomegalovirus promoter sequence, respectively.

Very recently, a further optimized 11 plasmid-only system-based reverse genetics system with increased virus generation efficiency was developed (Sánchez-Tacuba’s reverse genetics system) [30] (Figure 2). Sánchez-Tacuba’s reverse genetics system employs a chimeric helper expression plasmid encoding the African swine fever virus NP868R capping enzyme and T7 RNA polymerase under the control of the cytomegalovirus promoter [31], combined with a set of 11 rescue T7 plasmids encoding an RVA genome. In addition, genetically modified MA104 N*V cells are used for propagation of a small number of de novo synthesized recombinant RVAs, in which two components of the innate immunity system (interferon regulatory factor 3 (IRF3), and signal transducer and activator of transcription 1 (STAT1)) are degraded. Variations described pertain to the (over)expression of support proteins to enhance the recovery of recombinant RVAs. Sánchez-Tacuba’s reverse genetics system has enabled the recovery of simian strain RRV [32] and a murine-like RVA strain in addition to strain SA11-L2. The original 11 plasmid-only system and its variations have been exploited for the generation of reassortants [33,34,35,36,37], mutated viruses containing defined mutation(s) in the replicating genomes [38,39], and viruses expressing single or multiple reporters [29,30,40,41,42,43,44], proving the robustness of the original 11 plasmid-only system and 11 plasmid-only system-based procedures.

## 4. Entirely Plasmid-Based Reverse Genetics Systems for HuRVAs

Animal RVAs, such as simian SA11-L2 and RRV viruses, and HuRVAs share many features, but they differ in such as their host range, antigenic structures on outermost capsids, VP7 and VP4, and receptor-binding specificities.

RVAs are generally host restricted, although the interspecies transmission of RVAs with or without reassortment can occur in rare cases [45,46]. HuRVAs have human-specific G genotypes such as G1–G4, G9, and G12, and human-specific P genotypes such as P[4], P[6], and P[8], with very few exceptions [3]. In addition to the G and P genotypes, the other nine genes of HuRVAs also have distinct genotypes from those of animal RVAs [47]. Furthermore, HuRVAs use different receptors from animal RVAs, that is, ‘sialidase-sensitive’ animal RVAs bind to external sialic acid residues on glycans, whereas ‘sialidase-insensitive’ HuRVAs bind to internal sialic acid moieties on glycans or to modified sialic acid moieties in oligosaccharide structures, such as those present in the GM1 ganglioside and histoblood group antigens [48,49,50,51,52,53,54,55]. Furthermore, HuRVAs grow 1–3 logs less efficiently in cultured cells and exhibit >2-logs higher 50% diarrhea doses (DD_50_) in pup infection experiments than simian RVAs [36,56]. Last but not least, HuRVAs exhibit a significantly higher dependency for trypsin-like proteases for infectivity acquisition than simian RVAs [57,58]. Therefore, the development of a reverse genetics system for HuRVAs was required for proper understanding of HuRVA biology and pathology; however, no HuRVA reverse genetics system had been reported, despite extensive attempts in many laboratories worldwide.

The development of the first HuRVA reverse genetics system was reported by Komoto et al. (2019) [59]. As a HuRVA reverse genetics backbone, strain KU [60] was selected, it being genetically very similar to the representative reference HuRVA strain Wa [61]. Both the KU and Wa viruses have the principal and most important G1P[8] genotype [62,63], and KU virus has been studied well as to antigenic structure and used in many RVA laboratories in the world for extensive studies. In addition, KU virus has an extensive history of growth in cell culture. Although the complete nucleotide sequences of the KU genome determined by traditional Sanger sequencing had been deposited in the GenBank/EMBL/DDBJ data libraries, the authors performed deep sequencing using a next-generation sequencer, which allowed determination of highly reliable full-genome sequences of KU virus. The authors then prepared 11 rescue T7 plasmids encoding the individual 11 gene segments of KU virus.

To generate infectious recombinant KU virus entirely from plasmid cDNAs, BHK-T7 cells were transfected with a set of 11 rescue T7 plasmids encoding a genome of KU virus according to the 11 plasmid-only system (Figure 1). However, the generation of recombinant KU virus was unsuccessful in several trials. Then, the VP4 segment from simian SA11-L2 virus was employed in place of the original KU-derived VP4 segment, because it has been found that the VP4 gene is mainly responsible for the replication efficiency of RVAs [27,64]. An infectious recombinant KU-based mono-reassortant virus possessing the SA11-L2-derived VP4 segment (named rKU-VP4SA11 virus) could be readily recovered by using the 11 plasmid-only system. The viral growth and plaque size of rKU-VP4SA11 virus were as high and large as those of the wild-type SA11-L2 virus. These results reconfirmed that the VP4 gene segment is the key for high replication efficiency of animal RVAs, and indicated the competencies of the prepared rescue T7 plasmids for strain KU, other than the VP4 segment, to generate recombinant KU virus.

Because the functionalities of at least 10 rescue T7 plasmids for strain KU could be confirmed, the authors then modified the recovery procedure for recombinant HuRVAs for efficient multiplication of a small number of nascent recombinant KU viruses within the transfected BHK-T7 cells. As modifications, the authors employed the roller-tube culture technique and a 3-fold increased trypsin concentration in the maintenance medium for co-culture of transfected BHK-T7 cells and overlaid CV-1 cells, and a roller-tube culture with MA104 cells. These processes are beneficial for HuRVA propagation, and similar to the procedures for the isolation by cell culture of fastidious HuRVA strains from diarrheic stool specimens from RVA gastroenteritis patients. These modifications led to the successful recovery of infectious recombinant KU virus (named rKU virus). The rKU virus showed similar growth kinetics and plaque size to the native KU virus, demonstrating that the replication characteristics of rKU virus are indistinguishable from those of the native KU virus. Thus, the minor modifications raising the efficiency of isolation of a small number of recombinant HuRVAs from the transfected BHK-T7 cells were revealed to be the critical steps in the development of a HuRVA reverse genetics system.

**Figure 3:**
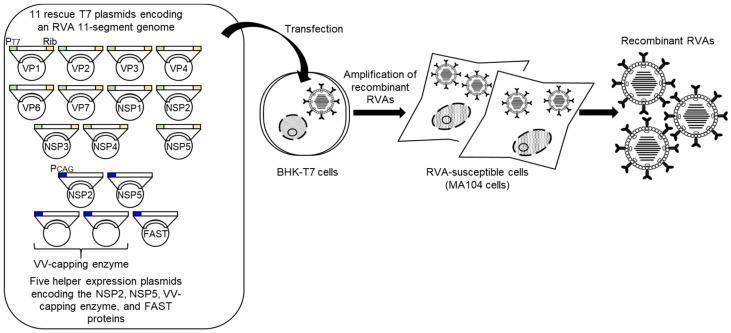
Schematic representation of Kawagishi’s reverse genetics system. BHK-T7 cells are transfected with a set of 11 rescue T7 plasmids encoding an RVA genome as well as five helper expression plasmids for the NSP2, NSP5, VV-capping enzyme, and small membrane fusion proteins. After 2 days, the transfected BHK-T7 cells are overlaid with RVA-susceptible cells (MA104 cells) and then co-cultured for 5 days for amplification of recombinant RVAs. Recombinant RVAs can be rescued from the co-cultures. PT7, Rib, PCAG, and FAST denote the T7 RNA polymerase promoter sequence, HDV ribozyme sequence, CAG promoter sequence, and small membrane fusion protein, respectively.

After this initial report of the development of a HuRVA reverse genetics system, the engineering of two other HuRVA strains was described [30,65]. Sánchez-Tacuba et al. (2020) [30] reported the artificial generation of a vaccine candidate HuRVA strain, CDC-9, possessing the G1P[8] genotype [66] using the Sánchez-Tacuba’s reverse genetics approach described above (see Section 3) (Figure 2), in which two rescue T7 plasmids encoding the NSP2 and NSP5 genes were included at 3-fold greater levels than the other nine rescue T7 plasmids. Generation of a recombinant CDC-9 virus with a limited growth history in cell culture (only 11 passages) reconfirmed the robustness of their optimized reverse genetics approach for recombinant HuRVA generation. In addition, a similar approach based on the overexpression of the NSP2 and NSP5 proteins has been reported [65]. The authors described that transfection of BHK-T7 cells with 11 rescue T7 plasmids for the genome of high-yield HuRVA strain Odelia with the G4P[8] genotype [67] along with two helper expression plasmids coding for the NSP2 and NSP5 proteins as well as three helper expression plasmids for the VV-capping enzyme and small membrane fusion protein brought about recombinant Odelia virus, despite the co-transfection of 16 plasmids (Figure 3). Thus, genetic engineering of HuRVA genomes has become as easy as with simian RVAs, and all the HuRVA reverse genetics systems involve the overexpression of NSP2 and NSP5 proteins. Although applications of these HuRVA reverse genetics systems have just been initiated, reverse genetics approach will be a powerful tool useful for obtaining a proper understanding of the molecular biology of HuRVAs in multiple aspects, the development of rationally designed safe and effective next-generation HuRVA vaccines, and for employing HuRVAs as expression viral vectors of intestine-specificity.

## 5. Reverse Genetics Approach for the Generation of RVA Mono-Reassortants

The segmented nature of the RVA genome allows for reassortment between/among two or more viruses within a co-infected cell, and this phenomenon has been harnessed to generate mono-reassortant viruses for determining the function(s) of a gene segment and their use as live-attenuated vaccines such as worldwide-used RotaTeq (Merck, Kenilworth, NJ, USA). The reassortment of RVAs can occur at a high frequency both in vitro [68,69,70,71,72,73,74,75,76,77,78,79,80] and in vivo [32,81]. A panel of mono-reassortants has been used to determine which gene segment is associated with a particular phenotype of parental viruses. For use in vaccines, live-attenuated RVAs need to be subjected to reassortment procedures to incorporate VP7 and VP4 gene segments of HuRVAs into the backbone of animal RVAs. So far, the preparation of mono-reassortants has been achieved by infecting cultured cells with two different RVA strains at a high multiplicity of infection, cloning of progenies by plaque formation from the lysates of infected cells, and examining genomic dsRNA profiles of cloned viruses by polyacrylamide gel electrophoresis (PAGE) analysis. These classical experimental processes must be repeated many times for the isolation of a target mono-reassortant virus. Therefore, these procedures are time-consuming and laborious, and sometimes it is very difficult to prepare the desired mono-reassortant because reassortment events in members of the family *Reoviridae* appear to be nonrandom [82,83,84,85], and there is genetic compatibility between the two parental RVA strains [86,87]. Reverse genetics could be an obvious method of choice, allowing desired genetic constellations of reassortants. Fukuda et al. (2020) [36] demonstrated that a panel of recombinant mono-reassortants with a HuRVA KU-derived gene segment in the backbone of simian SA11-L2 virus could be produced easily and more quickly by using a reverse genetics approach than the classical approach. Similarly, Kawagishi et al. (2020) [65] reported the preparation of a panel of SA11-L2-based recombinant mono-reassortants with a HuRVA Odelia-derived gene segment. These mono-reassortant panels can be employed for analyzing the functions of individual HuRVA gene segments, although as reciprocal combinations, panels of mono-reassortants in which there are individual SA11-L2-derived gene segments in the HuRVA backbones are also required for determination of the exact function of each HuRVA gene segment.

If a novel RVA strain possessing a new genotype emerges in humans, the VP7 and/or VP4 genes from the newly emerging RVA can be amplified by reverse transcription (RT)-PCR or quickly chemically synthesized, and then cloned into rescue T7 plasmids used for virus engineering by reverse genetics. The reverse genetics approach will speed up the preparation of possible vaccine seed viruses against newly emerging HuRVAs from 2–6 months to 2 weeks, bypassing the need to isolate and evaluate the reassortants. Moreover, compared to classical procedures for the preparation of mono-reassortants, a reverse genetics approach provides a direct and more reliable technique for the purpose.

## 6. Conclusions

Because HuRVA disease remains a major medical problem, generation of recombinant HuRVAs to control the disease is a long-awaited achievement for the clinically important viral pathogens in humans. HuRVA reverse genetics systems are highly valuable techniques that have the potential to increase our understanding of the molecular biology of HuRVAs and provide a new way of vaccine design to ultimately control HuRVA disease. That is, recombinant HuRVAs engineered in this way can be employed to address multiple issues regarding virus-host cell interactions during HuRVA replication, such as cell entry, uncoating, genome replication, gene transcription, packaging, maturation, and viral release. Although there are no antivirals against HuRVA disease currently, the identification of any amino acid sequence that interacts with a cellular factor could lead to the development of new antivirals. Currently licensed and used live-attenuated RVA vaccines are safe and efficacious, but one of the greatest concerns about the use of the live vaccines is their genetic stability and hence their reversion to pathogenic wild-type viruses. Clearly, reverse genetics technology can also be exploited to produce live-attenuated vaccine strains with several amino acid replacements and/or deletions in gene segment(s), making reversion to the wild-type virus highly unlikely. Moreover, recombinant HuRVAs can be generated from just 11 rescue T7 plasmids for an RVA genome without the need of any helper expression plasmid in the 11 plasmid-only system, which is possibly suitable for clinical research purposes as well as basic ones. On the other hand, reverse genetics approaches with increased efficiency of virus generation requiring helper expression plasmid(s) may be more suitable for basic research purposes. In any case, the future appears to be bright for progress in understanding the molecular mechanisms that regulate HuRVA replication and pathogenesis, and in developing next-generation vaccines to prevent HuRVA disease and rotaviral vectors of intestine-specificity. HuRVA reverse genetics will serve as a useful platform for a new generation of HuRVA investigations in both basic and clinical research areas.

## Data Availability

Not applicable.
